# The effect of virtual reality simulation on nursing students’ communication skills: a systematic review and meta-analysis

**DOI:** 10.3389/fpsyt.2024.1351123

**Published:** 2024-07-05

**Authors:** Mi-Kyoung Cho, Mi Young Kim

**Affiliations:** ^1^ Department of Nursing Science, Chungbuk National University, Cheongju, Republic of Korea; ^2^ College of Nursing, Hanyang University, Seoul, Republic of Korea

**Keywords:** virtual reality, simulation, nursing students, communication skills, meta-analysis virtual reality, meta-analysis

## Abstract

Virtual reality simulation (VRS) allows individuals to experience a sense of presence in a virtual environment, and it has been increasingly adopted as a novel teaching method in nursing education. VRS can have positive effects, such as patient safety, privacy assurance in healthcare, and enhanced interest and immersion in education. It is important to synthesize the results to date to determine if VRS has the potential to improve communication skills in practical nursing students. This systematic literature review and meta-analysis investigated the effect of VRS on the enhancement of communication skills among nursing students. The Preferred Reporting Items for Systematic Reviews and Meta-Analyses guidelines were employed for systematic review and meta-analysis. The following “PICO” details were considered: population—nursing students, intervention—VRS targeting communication skills enhancement, comparator—control groups without intervention or those undergoing general classes, and outcomes—communication skills. The search strategy yielded 301 results from nine databases, and 10 studies were selected for inclusion in our analysis. To calculate the overall effect sizes of the primary and secondary outcomes in the included studies, we used MIX 2.0 Pro (Version 2.0.1.6, BiostatXL, 2017) for the meta-analysis. The overall effect size for communication skills was 0.44, signifying a significant effect. A meta-regression analysis examining communication skills revealed significant results for the following factors: Fund (Ref.: No), outcome measurement time (Ref.: Immediately), outcome follow-up (Ref.: No), and debriefing (Ref.: No). Considering factors such as variations in sample size, research approaches, and the effects of independent studies on communication skills, this systematic literature review and meta-analysis suggests that VRS significantly improves nursing students’ communication skills overall.

**Systematic review registration:**

https://www.crd.york.ac.uk/prospero, identifier CRD42023439064.

## 1 Introduction

Various innovative approaches have been adopted to transform nursing education over the years, but the COVID-19 pandemic has led to the reduction or suspension of clinical practicum programs (1), which has prompted a transformation in the paradigm of nursing education. In response to these changing circumstances, nursing schools in South Korea and other countries have adopted virtual reality simulation (VRS) to provide learners with opportunities to experience clinical environments and patient interactions in VR settings. Even after the COVID-19 pandemic, efforts to develop and implement media-based immersive educational content have continued as nursing education has evolved from traditional face-to-face methods to remote learning (2). VR, an emerging technological advancement in recent years, enables users to experience real-world settings emulated in a VR environment (3) and has been widely utilized in education.

VR is defined as the use of partial immersion through a digital learning environment (e.g., computer, tablet, phone, screen) to foster a perceived lived experience for an intended outcome (i.e., learning, entertainment) (4). For this study’s purpose, VRS was defined as encompassing a range of technologies, including augmented reality (AR) and mixed reality (MR), for consistency of terminology. This study outlines the criteria for selecting studies, particularly focusing on the types of interventions considered in Virtual Reality Simulations (VRS) such as VR, AR, MR, Extended Reality, and the Metaverse. The scope is primarily centered on VR but remains broad to encompass various technologies. Additionally, the methodology involves setting up specific scenarios or situations, allowing students to engage actively within these simulated environments. The use of VRS in education offers several advantages: It enables gaining experience by transcending time and space restrictions, creating immersive experiences with enhanced learning effectiveness, allowing for remote learning, and providing learners with immediate feedback and interactive learning opportunities (5). Further, VRS has demonstrated positive effects on patient safety, privacy, engagement, and immersion (6). It is a means to build confidence through the repeated practice of nursing procedures commonly performed during clinical practicum (7).

Further, studies applying VRS in nursing education have examined its effects on emotional immersion, engagement, learning confidence, and satisfaction (8, 9). According to a study analyzing the utilization of VRS in nursing practicum courses in South Korea, the most frequently evaluated variable in VR programs was learning flow (11 studies), followed by program satisfaction, self-efficacy, clinical nursing skill performance, and critical thinking (nine studies each) (10). Additionally, previous studies on VRS among nursing students have assessed improvements in immersion, clinical skills, knowledge application, problem-solving abilities, and nursing performance (11–13). Improvement in communication skills is not a primary outcome measure mentioned in VRS education.

However, studies have suggested that VRS can promote a high level of empathy during avatar-based dialogue and interactive processes (14). A representative communication model is the SMCR model, which categorizes communication into four core elements: Source, Message, Channel, and Receiver (15). This model further elaborates on the components constituting each element. Simulation scenarios are particularly effective in enhancing communication skills, as they offer opportunities to customize and practice each of these elements in various ways. As a new simulation teaching strategy, VRS had been shown to improve interpersonal communication skills in a pilot study of nursing students in the early 2010s (16). A recent paper also demonstrated that VRS is non-inferior to live simulations for communication skills performance in medical and nursing students (17). In a review of VRS among nursing students, avatar simulations were effective in enhancing knowledge, decision-making skills, communication, problem-solving abilities, and leadership, as well as enabling learner-centered education by giving students the opportunity to provide care for avatar patients of diverse ages, races, and sexes (18). Therefore, as VRS is an evolving educational field, communication skills could be improved through learning via VRS.

Various forms of VRS are being implemented in nursing education. However, a systematic review and analysis are necessary to determine the effectiveness of VRS, specifically on communication skills, and to identify the specific factors that contribute to its effectiveness in nursing students. Systematic literature reviews and meta-analyses are valuable for synthesizing the results of included studies, considering variations in sample size, differences in research approaches, and potential intervention effects from independent studies. To date, there are only a handful of review articles that confirm the effectiveness of non-face-to-face training methods, including VRS, in improving communication skills (19), and more in-depth analysis of contributing factors is needed. Therefore, this systematic literature review and meta-analysis will be useful in evaluating the overall effects of VRS-based education on nursing students’ communication skills.

In this context, this study investigated the effects of VRS on improving communication skills (primary outcome) as well as knowledge, self-efficacy, critical thinking, teamwork, learning satisfaction, and confidence (secondary outcomes) in nursing students through a systematic review and meta-analysis, ultimately seeking to provide a foundational understanding of VRS.

## 2 Methods

### 2.1 Search strategy and data sources

An initial literature search was conducted by a librarian, and a second search was conducted by two researchers (Cho, M.-K. and Kim, M.Y.) with advice from a meta-analysis expert in nine electronic databases, including PubMed, Cochrane, Embase-Ovid, CINAHL (CINAHL Complete), WoS (Web of Science platform), Scopus, PQDT (ProQuest Dissertations & Theses Global), APA PsycArticles, and RISS (Research Information Sharing Service). Articles published in Korean or English on or before April 30, 2023, were searched. Two researchers and a librarian collaboratively designed the search terms and search strategy, and we used the following keywords for the search: “ nursing students(s)” “virtual reality” “augmented reality” “mixed reality” “extended reality” “metaverse” “communication”, using AND/OR operators to combine these terms. The search was conducted from June 23, 2023, to July 31, 2023, and the specific search strategy and search equations are shown in **Supplementary Tables 1** and **2**. The search protocol was registered on the PROSPERO International Prospective Register of Systematic Reviews (no. CRD42023439064; https://www.crd.york.ac.uk/prospero) on July 4, 2023. Data from the included studies were collected in accordance with the Preferred Reporting Items for Systematic Reviews and Meta-Analyses (PRISMA) guidelines (20).

### 2.2 Inclusion and exclusion criteria

A systematic literature search was performed using a Population–Intervention–Comparison–Outcome–Study Design (PICOS) framework appropriate for our study. The study findings were reported with reference to the PRISMA 2020 Checklist (retrieved from https://www.prisma-statement.org/prisma-2020-checklist on September 30, 2023). The inclusion criteria were as follows: The study population (P) comprised nursing students, and the intervention (I) involved VRS (VR, AR, MR, extended reality, metaverse). The control (C) was conventional learning other than VRS or no intervention, and the outcome (O) was communication skills as the primary outcome and knowledge, self-efficacy, critical thinking, teamwork, learning satisfaction, and confidence as secondary outcomes. If two or more measurements were taken after the intervention, the effect size for the intervention was calculated using the first measurement taken after the intervention. To accurately calculate effect sizes, only studies that presented the number of participants, mean, and standard deviation were included. The study design included randomized controlled trials (RCTs) and a quasi-experimental study as well as single group studies without a control group. The exclusion criteria were as follows: studies that included students from other majors, studies that used conventional simulation learning and not VRS, studies that did not report communication skills as an outcome measure, studies that were not published in Korean or English, and papers in which the full text was unavailable.

### 2.3 Data extraction

**Figure 1:**
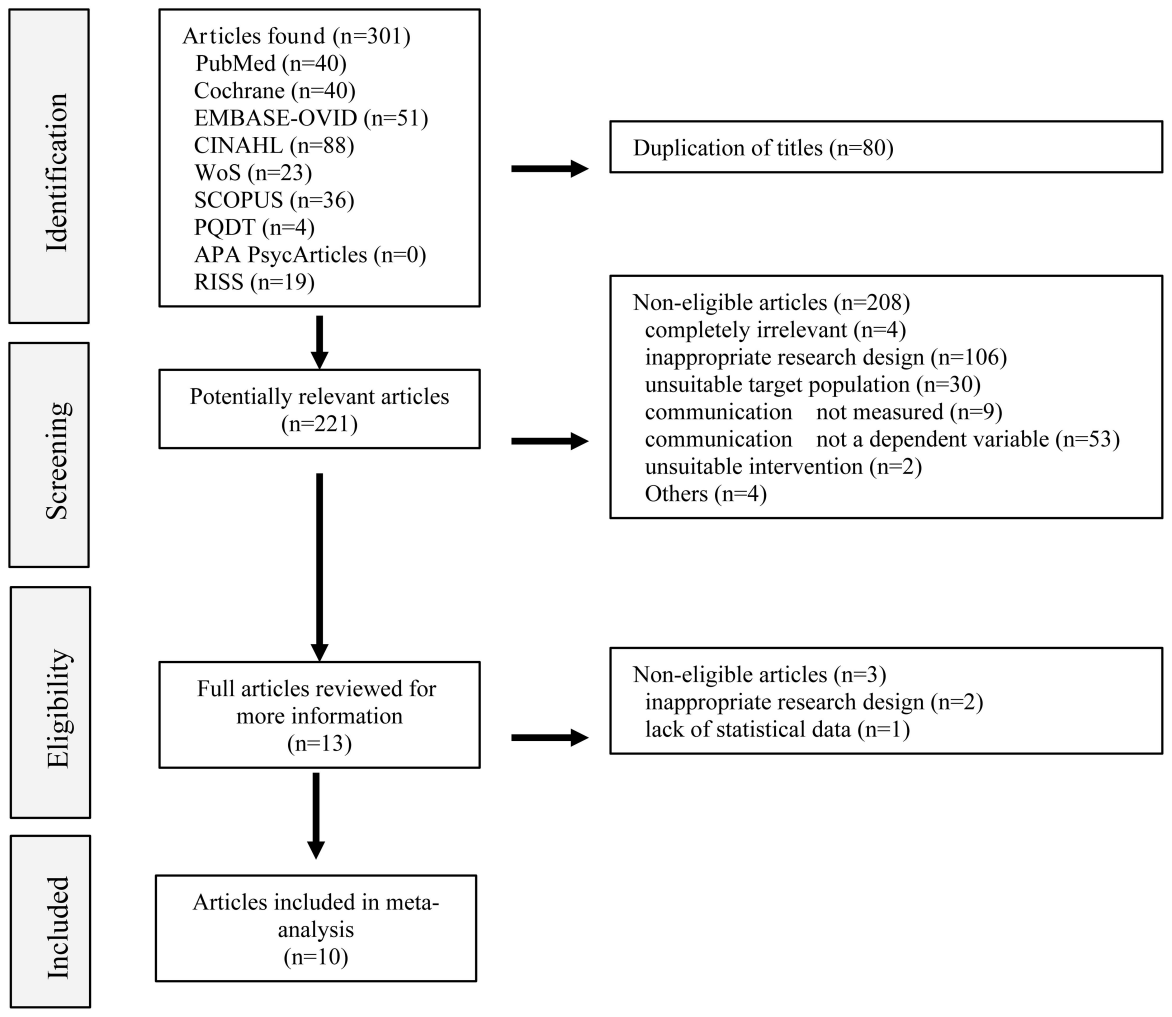
PRISMA flow diagram.

The researchers (Cho, M.-K. and Kim, M.Y.) organized the information of the selected studies using Microsoft Excel. They filtered the results by author, publication year, article title, and journal name, and removed duplicate records. The titles and abstracts of the studies were first reviewed against the inclusion and exclusion criteria, followed by a full-text review. Two literature searches were conducted independently (**Figure 1**). Eligible studies were selected, and data were extracted from the selected studies, including author information, publication year, country, institutional review board approval and funds, number of participants and brief characteristics, research design (RCT, Quasi-E, single group), intervention characteristics (intervention type, facilitator, intervention duration, intervention session, intervention operating time/session, pre-briefing, debriefing), dependent variables (Outcome measurement time, Outcome follow-up, communication as primary outcome variable, and other variables such as knowledge, self-efficacy, critical thinking, teamwork, and learning satisfaction and confidence), and quality scores in a coding book created using an Excel spreadsheet. The two researchers compared the coding results, and any discrepancies were resolved by reviewing the full text and reaching a consensus on the final coding value.

### 2.4 Statistical analyses

To combine the effect sizes of the primary and secondary outcomes in the included studies, MIX 2.0 Pro (Ver. 2.0.1.6, BiostatXL, 2017) was used for analysis. The effect sizes of individual studies and the overall effect were calculated using the random-effects model, which considers the heterogeneity among individual studies and adjusts the weights accordingly, and presented using Hedge’s g and 95% confidence intervals (CI). The weights for each effect size were determined using the inverse of variance (21). Heterogeneity among the included studies was assessed by calculating Higgins’ I^2^, which represents the proportion of actual variance relative to total observed variance (22). An I^2^ > 50% was considered to indicate heterogeneity (23). To explore the factors influencing the heterogeneity of primary outcomes, subgroup, meta-regression analysis, and exclusion sensitivity analyses were conducted based on the characteristics of the studies. In the meta-regression analysis, the independent variables consisted of characteristics of the studies that could contribute to heterogeneity, which were analyzed as dichotomous variables. Subgroup analyses were then conducted on these dichotomous variables to examine their potential as influencing factors in communication in VRS. Subsequently, simple regression analyses were performed to determine whether these dichotomous variables significantly influenced communication outcomes in VRS settings. Publication bias was assessed using funnel plots, trim and fill plots, Begg’s test, and Egger’s regression analysis after adjusting for the overall effect (24).

## 3 Results

### 3.1 Quality assessment

The quality of the included studies was appraised using the Joanna Briggs Institute (JBI) Checklist for RCTs and Checklist for Quasi-Experimental Studies (25, 26). The JBI Checklist for RCTs contains 13 items in the following domains: bias related to selection and allocation (three items); bias related to administration of intervention/exposure (three items); bias related to assessment, detection, and measurement of the outcome (three items); bias related to participant retention (one item); and statistical conclusion validity (three items) (25). The JBI Checklist for Quasi-Experimental Studies contains nine domains: cause and effect, baseline homogeneity testing, control over participants, use of a control group, measurement before and after intervention, description of dropouts, consistency of outcome measurement methods, and appropriateness of statistical analysis (26). Each item was rated using a system to assign 1 for “clear” and 0 for “unclear,” “no,” and “not applicable.” Three RCTs had a mean quality rating of 9.00, and all three RCTs had an unclear mention of “Q2. Was allocation to treatment groups concealed?” and “Q4. Were participants blind to treatment assignment?” Seven quasi-experimental studies were included, with a mean quality rating of 6.00. Six of these studies were single group studies given 0 for “Q2. Were participants included in any comparisons similar?” and “Q4. Was there a control group?” (**Table 1**).

**Table 1:** Quality assessment of the included studies.

Study ID	Joanna Briggs Institute of Critical Appraisal Tools Checklist for Checklist for Randomized Controlled Trials													Total score
	1	2	3	4	5	6	7	8	9	10	11	12	13	
3	1	0	1	0	0	0	1	1	1	1	1	1	1	9
4	0	0	1	0	1	1	1	1	1	1	1	1	0	9
7	1	0	1	0	0	0	1	1	1	1	1	1	1	9
Total	2	0	3	0	1	1	3	3	3	3	3	3	2	9.00

Study ID	Joanna Briggs Institute of Critical Appraisal Tools Checklist for Quasi-experimental Studies													Total score
	1	2	3	4	5	6	7	8	9					
1	1	1	1	1	1	1	1	1	1					9
2	1	0	1	0	1	0	1	1	0					5
5	1	0	1	0	1	0	1	0	1					5
6	1	0	1	0	1	0	1	1	1					6
8	1	0	1	0	1	0	1	1	1					6
9	1	0	1	0	1	1	1	1	1					7
10	1	0	1	0	0	1	1	0	0					4
Total	7	1	7	1	3	3	7	5	5					6.00

### 3.2 Characteristics of the included studies

The search strategy generated 301 results from nine databases, and after excluding duplicate results, 221 studies were identified. Thirteen studies were selected after the first round of review against the inclusion and exclusion criteria, and after reviewing the full texts, 10 studies were selected for our investigation. Six articles were published in or after 2022, and seven were published in Asia. Eight were approved by respective IRBs, and five were funded. Regarding study design, three were RCTs and seven were quasi-experimental studies. Six of them were single group studies, and five had a sample size of 60 or more. For the intervention, eight employed VR/AR simulation, and two used metaverse simulation. The facilitator was a faculty member in seven studies, and the intervention duration was four weeks or longer in three studies. The number of intervention sessions was eight weeks or longer in two studies and the intervention time per session was 1 h or longer in seven studies. Six studies used pre-briefing and eight studies used debriefing. Eight studies measured the dependent variable immediately after the intervention, whereas two measured the long-term effects. The quality rating was average or higher in five studies (**Table 2**).

**Table 2:** Descriptive summary of the included studies.

Study ID	Author (s) (year)	Country	IRB	Fund	Research design	Participants	Single group	Intervention type	Facilitator	Intervention duration	Intervention session	Intervention session time	Outcome measurement time	Outcome follow-up	Outcome variable	Pre-briefing	Debriefing	Quality score
1	Yang and Kang (27)	South Korea	Yes	No	Quasi	58 third-year nursing students (E: 29, C: 29)	No	Metaverse-based simulation	Researcher	3 weeks	3 sessions	100 min	Delayed (1 week)	None	Communication ability Knowledge Self-efficacy Critical thinking Learning satisfaction and Confidence	Yes	Yes	9
2	Liaw et al. (28)	Singapore	Yes	Yes	Quasi	32 students undertaking their final year of nursing courses (24 women)	Yes	Virtual-reality simulation	Researcher	None reported	None reported	2 h	Immediately	None	Communication knowledge Interprofessional communication self-efficacy	None	Yes	4
3	Silva et al. (29)	Spain	Yes	No	RCT	100 first-year students from the Faculty of Nursing, Physiotherapy, and Podiatry (E: 50, C: 50)	No	Virtual-reality simulation	Nursing faculty	None reported	None reported	None reported	Delayed (2 months)	None	Communication skill	None	None	9
4	Liaw et al. (17)	Singapore	Yes	Yes	RCT	120 undergraduate medical and nursing students (E: 60, C: 60)	No	Virtual-reality simulation	Nursing faculty	None reported	None reported	15 to 20 min/each scenario	Immediately	After 2 months	Team communication scale Teamwork attitudes	Yes	Yes	9
5	Traister (30)	USA	Yes	No	Quasi	33 nursing students recruited from two accredited registered nursing education programs	Yes	Virtual-reality simulation	Researcher	Session 1 occurred during weeks 4–7 of the semester Session 2 occurred during weeks 8–11	2 sessions	15 to 20 min	Immediately	After Session 2	Communication skills Anxiety	Yes	Yes	5
6	Song (31)	South Korea	No	No	Quasi	118 third-year nursing students (99 women)	Yes	Virtual-reality simulation	Nursing faculty	10 days	10 sessions	8 h/day	Immediately	None	Communication competence Social and Emotional competence Counseling self-efficacy	None	Yes	5
7	Sumi (32)	South Korea	Yes	No	RCT	100 second-year nursing students (E: 50, C:50)	No	Virtual-reality simulation	Nursing faculty	8 weeks	8 sessions	2 h	Immediately	None	Communication Academic Success Problem-solving in education	Yes	None	9
8	Kang and Moon (33)	South Korea	No	Yes	Quasi	54 third-year nursing students (46 women)	Yes	Metaverse-based simulation	Nursing faculty	None reported	None reported	220 min	Immediately	None	Communication Problem-solving process Learning self-efficacy	Yes	Yes	5
9	Kim and Chun (34)	South Korea	Yes	Yes	Quasi	82 second-year nursing students (62 women)	Yes	Virtual-reality simulation	Nursing faculty	5 weeks	4 sessions	2 h (sessions 1–3), 4 h for last session	Immediately	None	Communication skill Empathy Attitude of patient safety management Confidence in nursing care Clinical skill performance	None	Yes	7
10	Foronda et al. (35)	USA	Yes	Yes	Quasi	8 baccalaureate level students enrolled in the online Career Pathways course	Yes	Virtual-reality simulation	Nursing faculty	None reported	None reported	1 1/2 to 2 h (including debriefing)	Immediately	None	Communication performance	Yes	Yes	4

### 3.3 Effect of VR simulation-based interventions on communication skills

**Figure 2:**
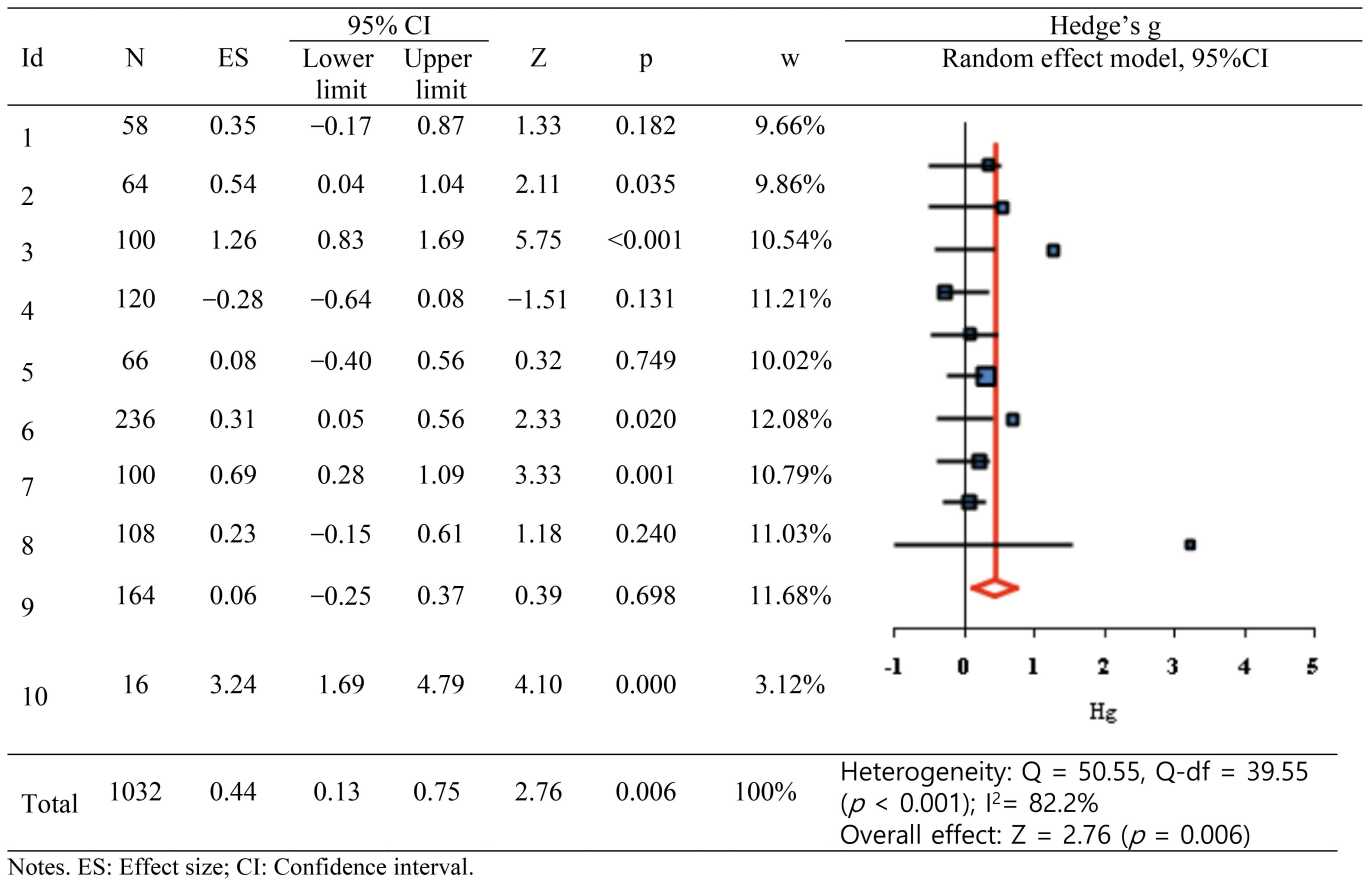
Effects of virtual reality simulation-based interventions on communication.

The overall effect of VRS on the primary outcome (communication skills) was Hedge’s g of 0.44 (95% CI: 0.13–0.75), indicating a moderate effect per the criteria proposed by Brydges (36) (**Figure 2**). Higgins’ I^2^ was 82.2%, indicating high heterogeneity. Subgroup analysis and meta-regression analysis were performed to identify the factors contributing to this heterogeneity.

The subgroup analysis showed that communication skills significantly improved in studies published in or after 2022 (Hedge’s g = 0.41, 95% CI: 0.04–0.78), studies not funded (Hedge’s g = 0.54, 95% CI: 0.15–0.92), quasi-experimental studies (Hedge’s g = 0.34, 95% CI: 0.06– 0.62), single group studies (Hedge’s g = 0.35, 95% CI: 0.02–0.68), studies with a sample size smaller than 60 (Hedge’s g = 0.51, 95% CI: 0.03–1.00), studies that used a VR/AR simulation-based intervention (Hedge’s g = 0.50, 95% CI: 0.11–0.89), studies that did not report an intervention duration or set the duration to less than four weeks (Hedge’s g = 0.55, 95% CI: 0.11–1.00), studies that had the intervention last 1 h or longer per session (Hedge’s g = 0.44, 95% CI: 0.14–0.73), studies that measured the outcome immediately after the intervention (Hedge’s g = 0.32, 95% CI: 0.02–0.62), studies that did not conduct follow-up measurements to shed light on the long-term effects of the intervention (Hedge’s g = 0.58, 95% CI: 0.24–0.92), studies that did not implement pre-briefing before the simulation (Hedge’s g = 0.52, 95% CI: 0.05–0.99), studies that did not implement debriefing after simulation (Hedge’s g = 0.97, 95% CI: 0.40–1.53), and studies with a below average quality assessment score (Hedge’s g = 0.46, 95% CI: 0.04–0.87). Additionally, the effect size differed significantly according to publication country, IRB, facilitator, and number of intervention sessions (**Table 3**).

**Table 3:** Subgroup analysis of communication by study characteristics.

Variables	Category	K	Study ID	n	ES	95% CI		Z	p
						Lower limit	Upper limit		
Year	<2022	4	4, 6, 7, 10	472	0.58	−0.07	1.23	1.74	.082
	≥2022	6	1, 2, 3, 5, 8, 9	560	0.41	0.04	0.78	2.19	.029
Country	Asia	7	1, 2, 4, 6, 7, 8, 9	850	0.25	0.02	0.48	2.14	.032
	Beyond Asia	3	3, 5, 10	182	1.30	0.07	2.53	2.07	.038
IRB	No	2	6, 8	344	0.28	0.07	0.49	2.59	.010
	Yes	8	1, 2, 3, 4, 5, 7, 9, 10	688	0.53	0.09	0.97	2.39	.017
Fund	No	5	1, 3, 5, 6, 7	560	0.54	0.15	0.92	2.74	.006
	Yes	5	2, 4, 8, 9, 10	472	0.35	−0.14	0.84	1.41	.158
Research design	Quasi-E	7	1, 2, 5, 6, 8, 9, 10	712	0.34	0.06	0.62	2.34	.019
	RCT	3	3, 4, 7	320	0.55	−0.35	1.45	1.20	.229
Participants	<60	4	2, 5, 8, 10	254	0.62	−0.02	1.27	1.89	.059
	≥60	6	1, 3, 4, 6, 7, 9	778	0.39	−0.01	0.78	1.93	.053
Single group	No	4	1, 3, 4, 7	378	0.50	−0.18	1.18	1.45	.146
	Yes	6	2, 5, 6, 8, 9, 10	654	0.35	0.02	0.68	2.10	.036
Intervention type	metaverse	2	1, 8	166	0.27	−0.03	0.58	1.74	.083
	VR/AR	8	2, 3, 4, 5, 6, 7, 9, 10	866	0.50	0.11	0.89	2.51	.012
Facilitator	Researcher	3	1, 2, 5	188	0.32	0.03	0.61	2.15	.031
	Nursing faculty	7	3, 4, 6, 7, 8, 9, 10	844	0.52	0.09	0.94	2.40	.016
Intervention duration	Not reported or <4 weeks	7	1, 2, 3, 4, 6, 8, 10	702	0.55	0.11	1.00	2.44	.015
	≥4 weeks	3	5, 7, 9	330	0.27	−0.14	0.68	1.31	.190
Intervention session	Not reported or < 8 sessions	8	1, 2, 3, 4, 5, 8, 9, 10	696	0.46	0.04	0.88	2.15	.032
	≥8 sessions	2	6, 7	336	0.46	0.10	0.83	2.47	.014
Intervention time/session	Not reported or <1 h	3	3, 4, 5	286	0.35	−0.59	1.29	0.73	.464
	≥1 h	7	1, 2, 6, 7, 8, 9, 10	746	0.44	0.14	0.73	2.91	.004
Outcome measurement time	Immediately	8	2, 4, 5, 6, 7, 8, 9, 10	874	0.32	0.02	0.62	2.07	.039
	Delayed	2	1, 3	158	0.82	−0.07	1.71	1.80	.071
Outcome follow up	No	8	1, 2, 3, 6, 7, 8, 9, 10	846	0.58	0.24	0.92	3.36	.001
	Yes	2	4, 5	186	−0.14	−0.48	0.20	−0.79	.431
Pre-briefing	No	4	2, 3, 6, 9	564	0.52	0.05	0.99	2.17	.030
	Yes	6	1, 4, 5, 7, 8, 10	468	0.40	−0.06	0.87	1.70	.089
Debriefing	No	2	3, 7	200	0.97	0.40	1.53	3.36	.001
	Yes	8	1, 2, 4, 5, 6, 8, 9, 10	832	0.26	−0.02	0.54	1.80	.073
Quality score	<Mean	5	2, 5, 6, 8, 10	490	0.46	0.04	0.87	2.16	.031
	≥Mean	5	1, 3, 4, 7, 9	542	0.41	−0.11	0.93	1.54	.124

K, number of analysis set; N, number of participants; ES, effect size; CI, confidence interval; IRB, institutional review board; Quasi-E, quasi-experimental study; RCT, randomized controlled trials.

The potential effects on the heterogeneity of effect sizes were analyzed using univariate meta-regression analysis by incorporating study characteristic variables that showed differences in the subgroup analysis. Communication skills were significantly influenced in studies that were not funded (*Z* = −2.97, *p* = .003), studies that did not measure the outcome immediately after the intervention (*Z* = 3.63, *p* <.001), studies that did not conduct follow-up measurements to shed light on the long-term effects of the intervention (*Z* = −3.57, *p* <.001), and studies that did not implement debriefing after the simulation (*Z* = −4.64, *p* <.001; **Table 4**).

**Table 4:** Meta-regression analysis evaluating communication.

Covariates (Ref.)	Estimate	SE	95% CI		Z	p
			Lower limit	Upper limit		
Year (Ref.: < 2022)	0.03	0.05	−0.06	0.12	0.58	.565
Country (Ref.: Asia)	0.61	0.17	0.27	0.96	3.51	<.001
IRB (Ref.: No)	0.07	0.13	−0.20	0.33	0.49	.621
Fund (Ref.: No)	−0.38	0.13	−0.63	−0.13	−2.97	.003
Research design (Ref.: Quasi-E)	0.20	0.14	−0.08	0.47	1.41	.158
Participants (Ref.: <60)	−0.03	0.15	−0.32	0.26	−0.19	.847
Single group (Ref.: Yes)	0.19	0.13	−0.07	0.45	1.40	.161
Intervention type (Ref.: metaverse)	0.06	0.17	−0.27	0.40	0.37	.708
Facilitator (Ref.: Researcher)	0.01	0.16	−0.31	0.33	0.06	.956
Intervention duration (Ref.: Not reported or <4 weeks)	−0.12	0.14	−0.38	0.15	−0.85	.393
Intervention session (Ref.: Not reported or <8 sessions)	0.14	0.14	−0.13	0.40	1.01	.315
Intervention time/session (Ref.: Not reported or <1 h)	0.05	0.14	−0.23	0.33	0.34	.730
Outcome measurement time (Ref.: Immediately)	0.66	0.18	0.30	1.02	3.63	<.001
Outcome follow up (Ref.: No)	−0.58	0.16	−0.90	−0.26	−3.57	<.001
Pre-briefing (Ref.: No)	−0.18	0.13	−0.43	0.07	−1.39	.164
Debriefing (Ref.: No)	−0.77	0.17	−1.09	−0.44	−4.64	<.001
Quality score (Ref.: < Mean)	0.00	0.13	−0.25	0.25	−0.03	.978

Ref., reference; SE, standard error; CI, confidence interval; IRB, institutional review board; Quasi-E, quasi-experimental study.

To verify whether individual studies affected the overall effect size, a meta-analysis was performed by sequentially excluding each study one by one, and the pooled effect size of VRS and its statistical significance were examined using an exclusion sensitivity test (37). Hedge’s g ranged from 0.32 to 0.52, indicating a small to moderate effect size, and the 95% CI (ranging from 0.04 to 0.21 and 0.59 to 0.86) did not include 0, signifying statistical significance in all cases. As it did not markedly differ from the effect size calculated based on all 10 studies (Hedge’s g = 0.44) and was significant, this meta-analysis was determined to be robust (**Table 5**).

**Table 5:** Exclusion sensitivity test of the virtual reality simulation-based intervention.

Study ID	K	ES	95% CI		Z	p
			Lower limit	Upper limit		
1	9	0.46	0.11	0.80	2.61	.009
2	9	0.44	0.09	0.78	2.50	.012
3	9	0.32	0.04	0.59	2.26	.024
4	9	0.52	0.21	0.83	3.28	.001
5	9	0.49	0.15	0.83	2.79	.005
6	9	0.48	0.10	0.86	2.50	.012
7	9	0.42	0.08	0.76	2.40	.017
8	9	0.48	0.12	0.83	2.65	.008
9	9	0.50	0.15	0.85	2.78	.005
10	9	0.35	0.07	0.62	2.47	.013

K, number of analysis set; ES, effect size; CI, confidence interval.

### 3.4 Effects of the intervention program on secondary outcomes

Secondary outcomes were knowledge, self-efficacy, critical thinking, teamwork, learning satisfaction, and confidence, and only critical thinking was significantly affected. After VRS intervention, critical thinking significantly improved, showing a large effect size of Hedge’s g = 1.32 (95% CI: 0.25–2.39; **Table 6**).

**Table 6:** Effect of virtual reality simulation-based interventions on other variables.

Variables	K	Study ID	n	ES	95% CI		Z	p
					Lower limit	Upper limit		
Knowledge	3	1, 2, 7	222	2.72	−0.27	5.71	1.79	.074
Self-efficacy	3	1, 6, 8	402	0.08	−0.12	0.27	0.79	.428
Critical thinking	4	1, 7, 8, 9	428	1.32	0.25	2.39	2.42	.015
Teamwork	2	1, 6	356	0.20	−0.11	0.52	1.27	.203
Learning satisfaction and confidence	2	1, 9	222	2.00	−0.52	4.51	1.56	.119

K, number of analysis set; N, number of participants; ES, effect size; CI, confidence interval.

### 3.5 Publication bias

**Figure 3:**
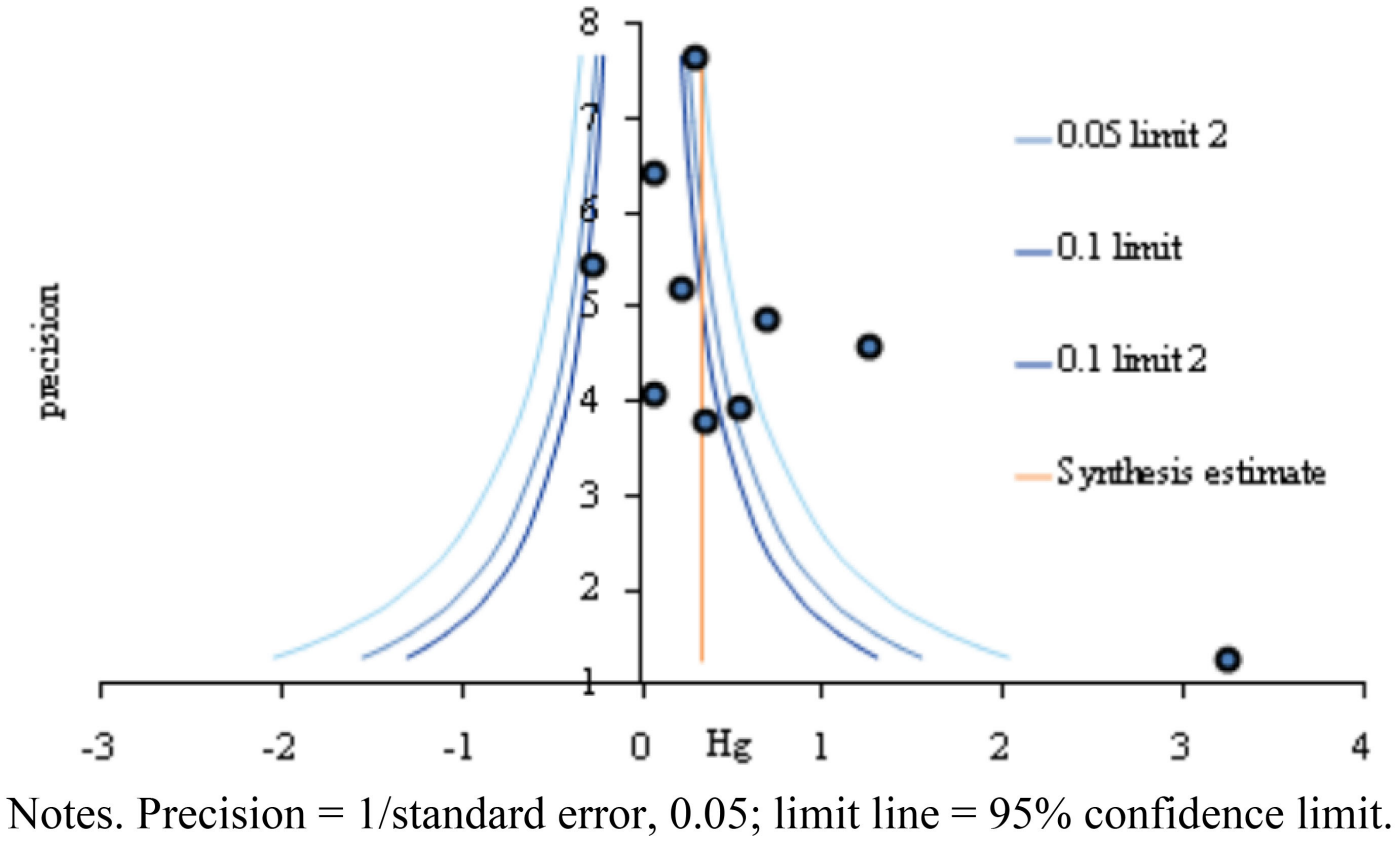
Funnel plot based on studies assessing virtual reality simulation-based interventions and their impact on communication.

**Figure 4:**
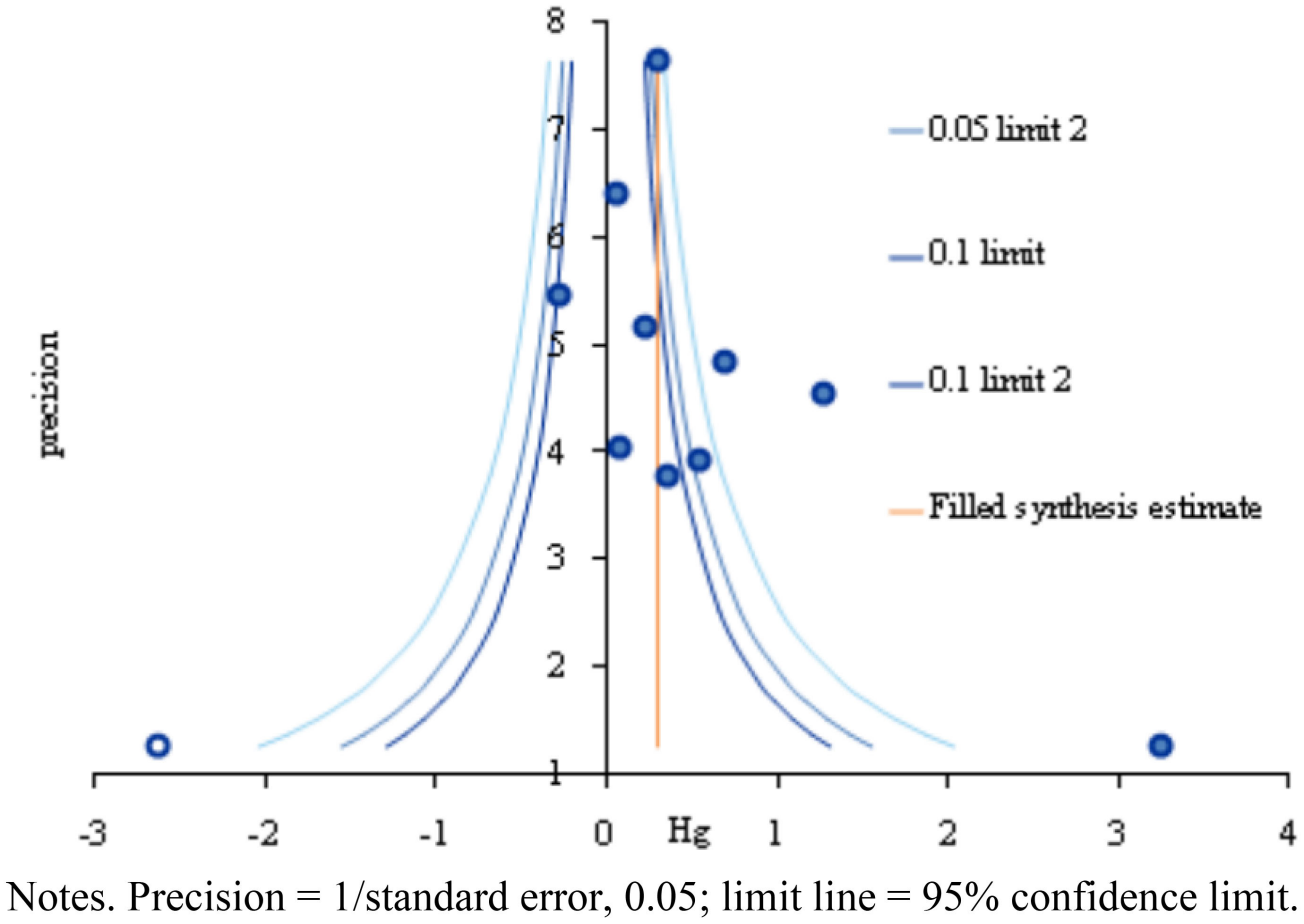
Trim and fill plot based on studies assessing virtual reality simulation-based interventions and their impact on communication.

Funnel plot and trim and fill plot analyses were conducted to assess publication bias. The individual effect sizes of the 10 studies included in this review (represented by blue circles) were slightly skewed to the right, indicating some degree of publication bias (**Figure 3**). Additionally, the trim and fill plot suggested the addition of one study (represented by a white circle) skewed to the left (**Figure 4**). Moreover, the trim and fill method (38) estimated that only one additional study needed to be included in the analysis. The adjusted effect size for the 11 studies was 0.35 (95% CI: 0.15–0.68). Although the effect size for communication skills decreased slightly after correction, it remained significant (**Table 7**).

**Table 7:** Publication bias test of virtual reality simulation-based interventions for communication.

Begg’s test	Tau b	K	S (P-Q)	ties	Z	p
Standard	0.38	10	17	0	1.52	.128
Corrected	0.36	10	17	0	1.43	.152
Egger’s regression test	Coefficient	SE	95% CI		Z	p
			Lower limit	Upper limit		
Intercept	3.87	2.04	−0.12	7.87	1.90	.057
Slope	−0.41	0.41	−1.21	0.39	−1.01	.314
Trim and fill method	K	ES	95% CI		Z	p
			Lower limit	Upper limit		
Original	10	0.44	0.13	0.75	2.76	.006
Corrected	11	0.35	0.15	0.68	2.05	.041

Begg’s test for rank correlation; Egger’s regression test for zero intercept; SE, standard error; CI, confidence interval; K, number of analysis set; ES, effect size.

## 4 Discussion

In our analysis, VRS significantly influenced communication skills with an effect size of 0.44. This result supports previous findings that simulation serves as a tool that can be safely used and one that provides students with opportunities to develop various skills, including priority setting and communication, in simulated cases of patient care in a clinical setting, by applying the theories that they have learned (39, 40). Further, as previously suggested, the implementation of VR allows participants to enhance their metacognition through spatial imagination via a virtual environment design, which enables them to perceive and individualize the physical environment in a clinical practicum and imagine virtual therapeutic relationships through the individual gestures of VR avatars and virtual patients (1). By experiencing scenarios that mimic real-life interactions in a virtual environment, they are able to react and receive real-time feedback, which seems to help them learn communication skills. These aspects suggest that VRS is an intervention that helps foster communication skills. Communication kills development by facilitating immersive, context-rich scenarios that closely simulate real-life interactions. Through role-playing diverse communicative functions in a variety of settings—such as the sender, receiver, and mediator of messages—students engage in dynamic exchanges that mirror authentic communicative challenges. Considering that practical education prioritizes skill acquisition, the incorporation of VRS creates environments where communication skills are not merely enhanced but are central to the learning objectives.

Notably, the following factors contributed to improving communication skills based on the results from the meta-regression analysis: First, by country, the effects on communication skills were significantly greater in studies conducted in Asia than in those conducted in other countries. This indicates that the impact of communication skills could vary depending not only on geographical context but also on cultural, contextual, and healthcare environmental aspects (41, 42). Moreover, in terms of funds, the effects on communication skills were significantly higher in studies that were not funded than in those that were funded. Interpreting the role of funding alone could be challenging, and it could be necessary to comprehensively examine this in conjunction with other variables to gain a better understanding of its impact.

Regarding the timing of outcome measurements, delayed measurements, as opposed to immediate ones, significantly enhanced communication skills. This observation aligns with the perspective of Cole and Bird (43), who emphasized the importance of systematic curriculum planning, effective teaching methods, and continuous feedback on students’ communication performance for the sustained development of communication skills over the medium to long term. Therefore, these results suggest that communication skills do not rapidly become sophisticated in a short time but, rather, take time to develop. Further, outcomes notably improved when follow-up assessments were conducted compared to cases in which no follow-up was performed. Follow-up assessments likely captured the impact of participants’ experiences after program completion, and further analysis is deemed necessary.

Regarding debriefing, not conducting it had a more significant positive impact on improving communication skills compared to when debriefing was conducted. This finding contrasts with the generally emphasized effectiveness and importance of debriefing in in-person simulations (44). Debriefing in in-person simulations typically involves team interactions, reflection, and discussions, whereas debriefing in a virtual setting is learner-driven, in which the learners themselves run the debriefing and receive individual feedback (45). As such, while VRS enables repeated and reflective learning through debriefing involving immediate tailored feedback to students (45), this could just be simple feedback that does not facilitate deep reflection. Therefore, the same effects of debriefing in in-person simulations cannot be obtained, and our results reflect this context.

The variables that did not have a significant impact on improving communication skills were as follows: Regarding the publication year, when categorized with a cutoff of 2020 and considering the relatively recent introduction of VRS, it had no impact on the improvement of communication skills. This suggests that while a growing number of simulation classes use clinical scenarios similar to actual clinical settings (1), implementing more recently developed technology does not seem to have a significant impact on communication skills.

Other factors, such as IRB approval and research design, were not significant. Further, the number of participants, use of a control group, and type of intervention in the control group were not significantly associated with communication skills. Similarly, intervention program-related variables, including the facilitator, intervention duration, and time per intervention session, did not significantly affect communication skills. There was no significant association between pre-briefing and quality scores. These results contrast with previous findings that pre-briefing is an important factor in in-person simulations, as it affects simulation preparation (46). This could be because to teach the simulation scenario, most of the included studies have mainly implemented pre-briefing as individual virtual activities, such as pre-learning or pre-quiz, rather than the diverse activities included in pre-debriefing for in-person simulations.

In addition to communication skills, critical thinking was another outcome variable that was significantly enhanced through VRS. This result is consistent with previous findings by which nursing students with better communication skills exhibit more critical thinking (47, 48). Communication skills encompass the ability to understand and interpret information, articulate it appropriately, and write effectively, as well as the ability to understand implications in symbols or behaviors used by others (49). In essence, it appears that both communication skills and critical thinking significantly improve as critical thinking during communication allows individuals to systematically analyze situations and evaluate them logically, thus enabling them to express their opinions in interpersonal relationships logically and clearly.

No significant differences were observed in knowledge, self-efficacy, teamwork, learning satisfaction, and confidence. As these variables are typically known to improve in team-based in-person simulation situations (50), our findings based on VRS are contradictory. The contrasting outcomes between virtual and face-to-face situations suggest that these two types of simulations have fundamentally different factors at play. Hence, further research, such as systematic reviews and meta-analyses, on the unique elements at play in VRS is recommended.

In in-person simulations, nursing students can learn accurate and effective communication skills through the use of role-plays and standardized patients in high-fidelity simulation scenarios, and communication among team members (17). However, in VRS, the key elements for improving communication skills are likely to be not adequately incorporated. This highlights the importance of using more detailed designs for VRS.

This study has several limitations. Firstly, The studies included in this review were quite heterogeneous, as they recruited students from a variety of grade levels. Students at some grade levels may not have had any experience with communication courses or clinical practice. This may have affected the degree of communication skills. Secondly, while the studies included in the analysis had in common the use of VRS, the programs were found to be different. (e.g., blended learning, role play, team-based learning). This may have contributed to the differences in effectiveness.

## 5 Conclusion

A meta-analysis investigated communication skill improvements in nursing students following VRS interventions. VRS notably enhanced communication skills with an effect size of 0.44, corroborating previous findings that simulations are effective, safe tools for developing a variety of skills, including priority setting and communication, through applied learning in simulated patient care scenarios. However, the study found no significant differences in outcomes concerning key operational aspects of the programs. This suggests that while VRS provides the benefits of a virtual environment, its impact on learners may differ from traditional face-to-face simulations. Thus, it is crucial to recognize that merely transferring the components of in-person simulations into a virtual setting does not automatically replicate effectiveness. The findings highlight the necessity for educational and research strategies specifically designed for the distinct dynamics of virtual environments, aimed not only at enhancing communication skills but also at improving other learner competencies.

## Data availability statement

The original contributions presented in the study are included in the article/**Supplementary Material**. Further inquiries can be directed to the corresponding author.

## Author contributions

M-KC: Conceptualization, Data curation, Formal analysis, Methodology, Project administration, Resources, Software, Validation, Visualization, Writing – original draft, Writing – review & editing. MK: Conceptualization, Data curation, Funding acquisition, Investigation, Resources, Supervision, Validation, Visualization, Writing – original draft, Writing – review & editing.
