# Supplementary material for: The effect of virtual reality simulation on nursing students’ communication skills: a systematic review and meta-analysis
Source: Front Psychiatry. 2024 Jul 5;15:1351123. doi: 10.3389/fpsyt.2024.1351123 (PMC11258010; doi:10.3389/fpsyt.2024.1351123)
Supplement: Supplementary file 1 [file Table_1.docx]

Supplementary Table 1. Search strategy

| Keyword | Search term | | | |
| --- | --- | --- | --- | --- |
|  | MeSH | PubMed Entry Terms | EMTREE (EMBASE) | Natural language |
| nursing student(s) | "Students, Nursing"[Mesh] | Pupil Nurses Student, Nursing Nurses, Pupil Nurse, Pupil Pupil Nurse Nursing Student Nursing Students | nursing student/ | ((student* OR pupil*) AND nurs*) |
| virtual reality | "Virtual Reality"[Mesh] | Reality, Virtual Virtual Reality, Educational Educational Virtual Realities Educational Virtual Reality Realtiy, Educational Virtual Virtual Realities, Educational Virtual Reality, Instructional Instructional Virtual Realities Instructional Virtual Reality Realities, Istructional Virtual Reality, Instructional Virtual Virtual Realities, Instructional | virtual reality/ | ((educational OR instructional) AND virtual realit*) |
| augmented reality | "Augmented Reality"[Mesh] | Augmented Realities Realities, Augmented Reality, Augmented Mixed Reality Mixed Realities Realities, Mixed Reality, Mixed | augmented reality/ | (augmented OR mixed) AND realit*) |
| mixed reality |  |  | - |  |
| extended reality | - |  | - | extended realit* |
| metaverse | - |  | - | metaverse OR meta-verse |
| communication | "communication"[Mesh] | personal communication communication, personal communication programs communication program program, communication programs, communication social communication communication, social communications, social social communications misinformation misinformations communications personnel personnel, communications miscommunication miscommunications | interpersonal communication/ | communication* |
|  | "Controlled Clinical Trials as Topic"[Mesh] | Clinical Trials, Controlled as Topic | controlled clinical trial (topic)/ OR Controlled Clinical Trials as Topic.mp. |  |

Supplementary Table 2. Search example on PubMed

| Search | Query | Search resutls |
| --- | --- | --- |
| #1 | "students, nursing"[MeSH] OR (("student*"[All Fields] OR "pupil*"[All Fields]) AND "nurs*"[All Fields]) | 73,210 |
| #2 | "virtual reality"[MeSH] OR (("educational"[All Fields] OR "instructional"[All Fields]) AND "virtual realit*"[TW]) | 6,519 |
| #3 | "Augmented Reality"[Mesh] OR (("augmented"[All Fields] OR "mixed"[All Fields]) AND "realit*"[TW]) | 6,423 |
| #4 | "extended realit*"[TW] | 206 |
| #5 | "metaverse"[TW] | 240 |
| #6 | #2 OR #3 OR #4 OR #5 | 12,357 |
| #7 | "Communication"[Mesh] OR "communication*"[TW] | 675,908 |
| #8 | #1 AND #6 AND #7 | 40 |
